# Detection of cerebral cavernous malformation associated developmental venous anomalies in gradient-echo and susceptibility-weighted magnetic resonance imaging: can we skip the contrast?

**DOI:** 10.1007/s00234-025-03666-2

**Published:** 2025-06-13

**Authors:** Laurèl Rauschenbach, Alejandro N. Santos, Jan Rodemerk, Hanah H. Gull, Thiemo F. Dinger, Adrian Engel, Maximilian Schüssler, Yan Li, Marvin Darkwah Oppong, Ramazan Jabbarli, Benedikt Frank, Michael Forsting, Karsten H. Wrede, Ulrich Sure, Philipp Dammann

**Affiliations:** 1 Department of Neurosurgery and Spine Surgery, University Hospital Essen, Hufelandstrasse 55, 45147 Essen, Germany; 2 Center for Translational Neuro- and Behavioral Sciences, C-TNBS, University Duisburg Essen, Essen, Germany; 3 Institute of Diagnostic and Interventional Radiology and Neuroradiology, University Hospital Essen, Essen, Germany; 4 Department of Neurology, University Hospital Essen, Essen, Germany

**Keywords:** Cerebral cavernous malformation, Developmental venous anomaly, Magnetic resonance imaging, Gradient echo T2*-weighted imaging, Susceptibility weighted imaging

## Abstract

**Purpose:**

Hemosiderin-sensitive MRI sequences are commonly utilized for microbleed identification in cerebral cavernous malformations (CCM). Efficacy of susceptibility-weighted (SWI) and gradient-echo T2*-weighted imaging (GRE-T2*) sequences in detecting CCM-associated developmental venous anomalies (DVA) remains uncertain.

**Methods:**

We conducted a retrospective review of our institutional CCM database. Inclusion criteria comprised baseline characteristics and MRI datasets involving contrast-enhanced T1 (CE-T1), T2, and SWI or GRE-T2* sequences. The presence of CCM-related DVA was determined utilizing CE-T1 imaging. A subgroup of 200 patients, evenly distributed with or without DVA, underwent random selection for analysis, with 50 patients each having SWI and GRE-T2* imaging. The presence of DVA was evaluated by two blinded neuroradiologists based on SWI or GRE-T2* sequences. Interrater agreement, sensitivity, and specificity values for SWI and GRE-T2* sequences were analyzed.

**Results:**

Imaging assessment demonstrated observer agreement in 76% of the SWI sequences (K = 0.51, *p* <0.001) and 82% of the GRE-T2* images (K = 0.39, *p* <0.001). While SWI sequences exhibited a sensitivity of 81.4% and a specificity of 60.6%, GRE-T2* sequences showed a sensitivity of 19.1% and a specificity of 97.5%. Misdiagnoses were frequent in small vessel DVAs, whereas large vessel DVAs were associated with higher diagnostic accuracy in both SWI and GRE-T2* sequences (*p* =0.002).

**Conclusion:**

Evaluation of SWI and GRE-T2* sequences for assessing CCM-related DVA appears less effective than the routine use of CE-T1 sequences. The use of contrast agents still appears necessary for detailed diagnostics and appropriate surgery planning.

**Supplementary Information:**

The online version contains supplementary material available at 10.1007/s00234-025-03666-2.

## Introduction

Cerebral cavernous malformations (CCMs) are low-flow vascular lesions that have the potential to cause intracerebral hemorrhage (ICH). Occasionally, CCM may be accompanied by a developmental venous anomaly (DVA). These lesions are typically benign neurovascular variants but may present a risk for CCM removal as they can complicate surgical access and may cause severe venous bleeding or consequences of venous ischemia [3]. Consequently, preoperative management routinely involves screening for DVAs to evaluate surgical risk and determine optimal strategies [8]. Furthermore, in cases of multiple CCMs, the presence of a DVA may indicate whether the condition is familial or sporadic, as familial forms rarely exhibit associated DVAs, simplifying diagnostic pathways [22].

Currently, contrast-enhanced (CE) MRI is considered the gold standard for detecting both CCMs and associated DVAs [3]. Despite the utility of traditional T1- and T2-weighted sequences, employing T2-weighted gradient-echo (GRE-T2*) or susceptibility-weighted imaging (SWI) can enhance the identification of CCMs and DVAs [14, 18]. However, due to concerns regarding the cerebral deposition of gadolinium, a commonly used contrast agent with potential neurotoxic effects, research is increasingly exploring alternatives for contrast-enhanced examinations [17]. Moreover, gadolinium-based contrast agents are contraindicated for individuals with a history of anaphylactoid reactions, severe renal impairment, or during pregnancy [5]. Consequently, there is growing interest in non-contrast MRI techniques, particularly those capable of providing information on intracerebral structures without the need for contrast agents, and artificial intelligence-assisted processing techniques are being explored to enhance the diagnostic capabilities of these sequences [20, 21]. In this study, we investigate the efficacy of GRE-T2* and SWI imaging without the use of contrast-enhanced T1 (CE-T1) or T2 imaging in identifying DVAs coexisting with CCMs.

## Materials and methods

### Study design

The data of this study was derived from an institutional database and was retrospectively analyzed following the principles expressed in the Declaration of Helsinki. All data were derived from a national high-volume tertiary center with dedicated neurosurgical and neuroradiological experience in CCM disease. All procedures were approved by an institutional review board (identification board numbers #14–5751-BO and #19–8662-BO). Informed consent was obtained from all subjects and in the case of individuals younger than 18 from their legal guardians. Patients were eligible for study inclusion if they were treated in our tertiary care center between 2003 and 2023 and fulfilled the below-mentioned criteria.

### Inclusion criteria

Study inclusion required the following criteria: imaging-based diagnosis of CCM, no history of neurosurgical procedures or intracranial pathology, clinical baseline characteristics, and MRI dataset with thin-sliced multiplanar contrast-enhanced T1 (slice thickness: 1 mm), axial and sagittal T2 (slice thickness: 1–5 mm), and axial SWI (slice thickness: 1–5 mm) or axial GRE-T2* (slice thickness: 1–5 mm) sequences. Imaging was performed on 1.5 T or 3.0 T MRI machines. Patients with sporadic and familiar cavernomatosis were included.

### Data selection

Based on the institutional database and after applying the above-mentioned inclusion criteria, a subset of CCM patients with or without associated DVA was extracted (LR, PD). In case of disagreement, these cases were excluded from further analysis. Individuals with SW or GRE-T2* imaging were selected from the remaining cases. The list was fed to the online platform “Research Randomizer” (available at: www.randomizer.org) and 200 patients with DVA and SWI, without DVA and SWI, DVA and GRE-T2*, and without DVA and GRE-T2* were randomly selected, with 50 patients in each group. In the case of multiple CCMs, one lesion was randomly selected by the first author for subsequent analysis. Diagnosis of CCM included the presence of lesions with a reticulated core of mixed signal intensity on T2-weighted MRI and a hemosiderin rim in the surrounding brain parenchyma on hemosiderin-sensitive MRI sequences, with minimal or no enhancement in T1-weighted imaging [23]. Diagnosis of DVA required the presence of a white matter lesion, typically stellate or linear, converging into a collecting vein and draining into the dural, sinus, or deep veins, and an umbrella or caput medusa-like appearance on contrast-enhanced T1-weighted imaging [13]. The coexistence of CCM and DVA was proposed if the CCM was localized in the DVA draining territory [24]. Finally, two blinded, experienced, board-certificated, and independent neuroradiologists received SW or GRE-T2* images and needed to state on the presence or absence of DVA based on the hemosiderin-sensitive sequences.

### Data collection

Medical records and imaging data were used to assess clinical features, i.e., age, sex, CCM multiplicity, CCM localization, and lesion volume. Two independent authors conducted data extraction. Multiplicity was suggested in the case of > 1 CCM. Lesions were defined as deep-seated if located subcortically and below the deepest adjacent sulcus with a minimum distance from bony structures > 1 cm. All other lesions were defined as superficial. In the case of brainstem localization, all lesions were defined as deep-seated. Axial and sagittal images were used to calculate the largest craniocaudal (d_cc_), anteroposterior (d_ap_), and lateral (d_l_) diameter (in cm) of each lesion or the respective hemorrhage (LR, JR). Diameter-based volume (V) of each CCM was assessed (in cm^3^) according to the ellipsoid formula: V = d_cc_ x d_ap_ x d_l_ x π/6. Multiplanar contrast-enhanced T1-weighted images were used as DICOM dataset for subsequent image analysis using the software 3D-Slicer to assess the largest diameter of DVA and to allow classification in “small vessel DVA” (≤ 1.5 mm) and “large vessel DVA” (> 1.5 mm) (LR, AE). The magnetic field strength was identified from the DICOM report of each MRI examination.

### Statistical analyses

Statistical testing was performed using SPSS 27.0 and results were visualized using PRISM 9.0. Results were considered statistically significant at an alpha level of less than 0.05. Data were tested for normal distribution by performing a Shapiro-Wilk test. Data were expressed as the absolute number and valid percent or mean value and standard deviation. For univariate analysis with categorical variables, the chi-square (sample size > 5) or Fisher’s exact test (sample size ≤ 5) was applied. For univariate analysis with metric variables, the Student’s t-test was used. Cohen’s kappa was determined to assess the inter-rater reliability.

## Results

### Patient cohort

**Fig. 1:**
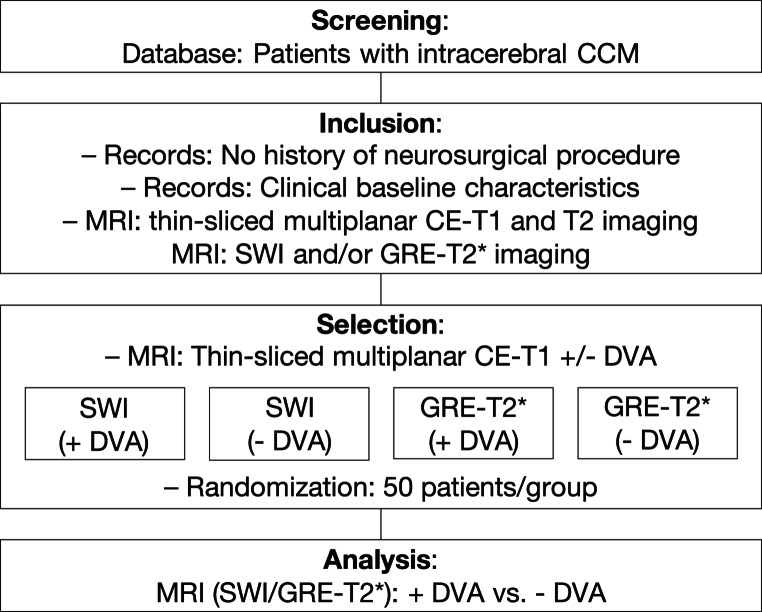
Flowchart of the study design. The principles of study inclusion and study conduct are shown

After reviewing the inclusion criteria, 200 patients were randomly selected to participate in the study. The selection process is described in Fig. 1. SWI sequences from one-half of the cohort and GRE-T2* sequences from the other half were analyzed. In each group, 50 patients had a CCM-associated DVA in the CE-T1 sequences, whereas the other 50 patients had no DVA. Almost half of the patients were female (54.0%) and the average age at the time of MRI was 43.2 ± 14.8 years. Of the 200 patients examined, most (77.5%) underwent imaging with a 1.5 T MRI, while a smaller proportion (21.5%) were imaged with a 3.0 T MRI. Information was unavailable for two patients (1%). The majority of subjects revealed singular CCMs (71.5%), while a few patients showed multiple CCMs (28.5%). The cohort showed a predominance of CCMs in the brainstem (21.5%), followed by CCMs in the frontal lobe (21.0%) and temporal lobe (18.5%). No predilection for one of the cerebral hemispheres existed. When DVA was present, it was usually of large caliber (60.0%), whereas some patients had a narrow vessel diameter (40.0%). Overall, the average volume of the investigated lesions was 5.14 (± 4.52) mm. In general, the lesions were deeply located (72.5%). Comparative analyses showed no differences in the distribution of traits among patients with SWI or GRE-T2* imaging. The characteristics are presented in Table 1. A comparison between patients with and without DVA showed that DVA is more common in infratentorial (*p* =0.01) and deep (*p* <0.001) CCMs. Comprehensive data are presented in Supplemental Table 1.

**Table 1:** Characteristics of patients and cavernomas. Abbreviations: CCM, cerebral cavernous malformation; CI, confidence interval; N, number of patients; N/A, not applicable; no, number; OR, odds ratio; SD, standard deviation; SWI, susceptibility-weighted imaging; GRE-T2*, gradient-echo T2* weighted imaging. Annotations: A, t-test; B, chi-square test or fisher test, where applicable; C, note that cavernomas of the midline are not considered here due to their rarity

	SWI	GRE-T2*	*p*	OR	95% CI
No of patients (N, %)	100 (100.0)	100 (100.0)	N/A	N/A	N/A
Age (years; mean ± SD)	42.2 (± 17.0)	44.1 (± 12.4)	0.35 ^A^	N/A	N/A
Sex (N, %)			0.48 ^B^	0.79	0.45–1.37
− Female	57 (57.0)	51 (51.0)			
− Male	43 (43.0)	49 (49.0)			
CCM multiplicity (N, %)			0.35 ^B^	1.40	0.76–2.62
− 1 CCM	68 (68.0)	75 (75.0)			
− ≥ 2 CCM	32 (32.0)	25 (25.0)			
CCM (cm^3^; mean ± SD)	4.8 (± 3.2)	5.5 (± 5.5)	0.33 ^A^	N/A	N/A
CCM side ^C^ (N, %)					
− Left	49 (53.3)	48 (49.0)	0.57 ^B^	1.19	0.67–2.10
− Right	43 (46.7)	50 (51.0)			
CCM localization (N, %)					
− Frontal lobe	20 (20.0)	22 (22.0)	0.86 ^B^	0.89	0.45–1.75
− Temporal lobe	18 (18.0)	19 (19.0)	0.99 ^B^	0.94	0.46–1.91
− Parietal lobe	6 (6.0)	10 (10.0)	0.44 ^B^	0.57	0.20–1.65
− Occipital lobe	5 (5.0)	8 (8.0)	0.57 ^B^	0.61	0.19–1.92
− Basal ganglia/thalamus	10 (10.0)	7 (7.0)	0.61 ^B^	1.48	0.54–4.05
− Ventricular system	1 (1.0)	1 (1.0)	0.99 ^B^	1.00	0.06–16.21
− Cerebellum	14 (14.0)	16 (16.0)	0.84 ^B^	0.86	0.39–1.86
− Brainstem	26 (26.0)	17 (17.0)	0.17 ^B^	1.72	0.86–3.41
CCM depth (N, %)			0.75 ^B^	1.16	0.62–2.16
− Superficial	26 (26.0)	29 (29.0)			
− Deep	74 (74.0)	71 (71.0)			
Vessel diameter (N, %)			0.31 ^B^	0.61	0.27–1.36
− Narrow	17 (34.0)	23 (46.0)			
− Large	33 (66.0)	27 (54.0)			

### Rating and interrater agreement

The evaluation of the blinded investigators showed agreement in 76% of the SWI sequences, while there was disagreement in 24% of the cases. This results in a Cohen’s Kappa value of Κ = 0.51 (*p* <0.001) for the SWI sequences, indicating a moderate observer agreement [15]. For the GRE-T2* sequences, there was agreement in 82% and disagreement in 18% of cases. This results in a Cohen’s Kappa value of Κ = 0.39 (*p* <0.001) for the SWI sequences, indicating a fair observer agreement [15]. Detailed data on the specific rating results are presented in Supplementary Fig. 1.

### Diagnostic test accuracy of SWI/GRE-T2* imaging

**Fig. 2:**
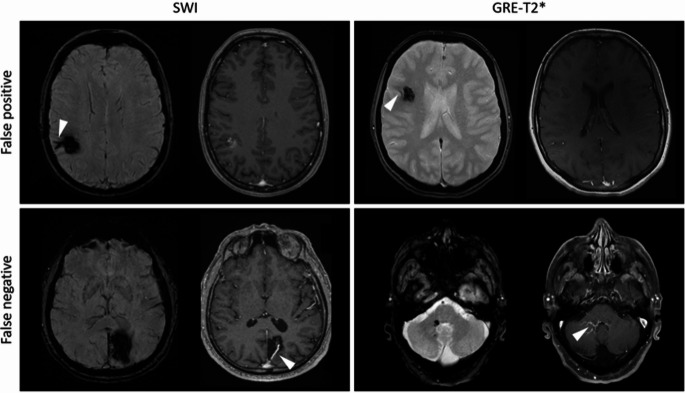
Exemplary cases of misdiagnosis. Axial MRI images are shown. SWI or GRE-T2* sequences are shown, next to the CE-T1 sequences of the same slice. The existing or suspected DVA is highlighted with an arrow

**Fig. 3:**
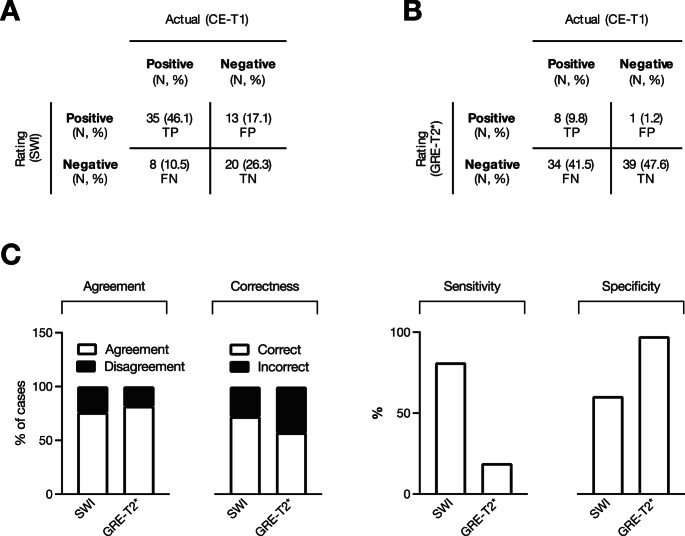
Rating results of SWI and GRE-T2* sequences. Four-field tables for the rating results based on the (**A**) SWI and (**B**) GRE-T2* sequences. For each of the two sequences, the true positive (TP), false positive (FP), true negative (TN), and false negative (FN) results are displayed as an absolute number of cases and as a relative number in percent. (**C**) The relative frequencies of agreement/disagreement and correct/incorrect assessments are shown, as well as the sensitivity (TP/TP + FN) and specificity (TN/TN + FP) of the respective test method

For further analyses, only those cases were used in which agreement could be achieved. The true positive (TP), false positive (FP), true negative (TN), and false negative (FN) results were calculated for both sequences. Exemplary cases for all four scenarios are shown in Fig. 2, both for the SWI and the GRE-T2* images. The four-field tables for the respective MRI sequences can be seen in Fig. 3A and B, respectively. From this, the sensitivity and specificity parameters could be calculated to make statements about the test accuracy. The SWI sequence showed a high sensitivity of 81.4% and thus a high degree of certainty for excluding DVA. The specificity, on the other hand, was moderate at 60.6%, meaning that the SWI sequence provided limited certainty for detecting DVA. Among patients with a DVA, 18.6% of cases yielded false-negative results, while among those without a DVA, 39.4% showed false-positive findings. This was different for the GRE-T2* sequence. It showed a low sensitivity of 19.1% but a high specificity of 97.5%. In the group of patients with a DVA, false-negative results occurred in 2.5% of cases, while false-positive findings were observed in 81.0% of cases. Detailed data is presented in Fig. 3C.

### Predictors of (mis)diagnosis with SWI/GRE-T2* imaging

It has been hypothesized that certain anatomical features correlate with the accuracy of a DVA diagnosis. This concerned the size of the DVA, as larger vessels are more likely to be detected than smaller ones, as well as the depth of the CCMs in the brain, as proximity to bony structures of the skull can provoke artifacts in hemosiderin-sensitive sequences. Cases in which an agreement was reached between the raters were subjected to univariate analysis. The parameters depth of the CCMs in the brain and DVA diameter were checked for a link to correct or incorrect diagnoses. The results revealed that a larger vessel diameter was associated with a correct diagnosis (*p* =0.002), while the depth of the CCMs did not influence the correctness of the diagnosis (*p* =0.999). Data is illustrated in Table 2. It was further evaluated whether the magnetic field strength influenced the correct or incorrect identification of a DVA. In the 1.5 T MRI examinations, 61.6% were correctly identified and 38.5% were incorrectly identified, whereas in the 3.0 T MRI examinations, 77.5% were correctly identified and 22.6% were incorrectly identified (*p* =0.141).

**Table 2:** Predictors of (mis)diagnosis with SWI/GRE-T2* imaging

	True	False	*p*	OR	95% CI
No of patients (N, %)	43 (100.0)	42 (100.0)	N/A	N/A	N/A
CCM depth (N, %)					
− Superficial − Deep	7 (16.3) 36 (83.7)	7 (16.7) 35 (83.3)	0.999 ^B^	0.97	0.31–3.06
Vessel diameter (N, %)					
− Narrow − Large	11 (25.6) 32 (74.4)	25 (59.5) 17 (40.5)	0.002 ^B^	0.23	0.09–0.59

*Abbreviations*: *CCM* cerebral cavernous malformation, *CI* confidence interval, *N* number of patients, *no* number, *OR* odds ratio, *SWI* susceptibility-weighted imaging, GRE-T2* gradient-echo T2* weighted imaging. Annotations: B, chi-square test or fisher test, where applicable

## Discussion

The present study aimed to assess the effectiveness of SWI and GRE-T2* sequences in identifying CCM-associated DVAs without the use of CE-T1 imaging. Although the overall risk of spontaneous hemorrhage from either CCMs, DVAs, or DVA-associated CCMs is relatively low, accurate detection remains clinically relevant [12, 25]. This is particularly true in patients with multiple CCMs, where the presence of an associated DVA may inform the underlying etiology, or in those undergoing surgical resection, where preoperative knowledge of a DVA is critical to avoiding potentially severe complications. Our findings suggest that while SWI and GRE-T2* sequences offer some diagnostic value, they fall short of the accuracy achieved with CE-T1 imaging, particularly evident in the 1.5 T MRI. This has potential implications for neurovascular surgeons, particularly regarding surgical planning in cases where larger DVAs may impact access and the approach to CCM resection [7]. Numerous reports in the literature detail instances of fatal DVA-associated venous bleedings and predominantly infarctions during perioperative periods of CCM removal. Current understanding suggests that a synergistic effect of intrinsic hypercoagulability and surgical manipulation, involving stretching, compression, or occlusion of the DVA, leads to insufficient venous outflow, resulting in subsequent venous congestion and bleeding [2]. Consequently, a Delphi consensus recommends the preservation of DVAs during CCM resection to mitigate these risks [8]. Hence, it is of utmost importance for the surgeon to be fully informed on the presence, dimensions, and exact localization of a DVA during the surgical planning phase, highlighting the imperative reliance on accurate imaging techniques.

Our results revealed sensitivities of 81.4% for SWI and 19.1% for GRE-T2* sequences in detecting DVAs, with corresponding specificity values of 60.6% and 97.5%. These values align with a study by Young and colleagues, who investigated the sensitivity of SWI sequences in detecting pediatric DVAs and described a value of 86% [28]. Various working groups that have investigated the usefulness of SWI or GRE-T2* sequences concerning the detection of CCMs report a significantly better sensitivity of SWI sequences, in line with the detection of DVAs [9, 10, 26]. Higher values were reported by Abdelgawad and colleagues, who examined 29 DVAs using SWI sequences [1]. This discrepancy in findings could be attributed to differences in sample size, patient selection criteria, as well as variations in imaging protocols. Moreover, the sensitivity and specificity of certain MRI sequences may vary depending on the investigation of solitary DVAs or CCM-related DVAs. Concerning DVA detection, our findings underscore the appropriate utility of SWI sequences but indicate the limited applicability of GRE-T2* sequences. Regarding DVA exclusion, our results reveal moderate utility of SWI sequences but strong applicability of GRE-T2* sequences. Taken together, both imaging modalities fell short of providing comprehensive diagnostic accuracy, especially evident in cases of small vessel DVAs. This suggests that while hemosiderin-sensitive MRI may aid in identifying larger vessel DVAs, it seems less reliable for detecting smaller vessel anomalies. For neurosurgical procedures, knowledge of large-diameter DVAs is crucial, which supports the argument that susceptibility-weighted sequences may be suitable for preoperative planning. However, since DVAs are increasingly used for differentiating between sporadic and familial cavernomatosis, the MRI-based detection of small-diameter DVAs is equally important, where susceptibility-weighted sequences may not be appropriate.

Notably, the discrepancy in diagnostic accuracy between SWI and GRE-T2* sequences may be attributed to differences in imaging principles. SWI uses phase information for data post-processing and emphasizes susceptibility effects from blood products, providing enhanced contrast between hemorrhagic lesions and surrounding tissues [11, 19]. In contrast, GRE-T2* sequences mainly rely on T2* relaxation, which may be less sensitive to subtle hemorrhagic changes, particularly in smaller vessels [27].

Susceptibility-weighted imaging sequences vary between MRI vendors, particularly in phase processing and image reconstruction. In our study, all SWI data were acquired on Siemens systems using standardized post-processing. We acknowledge that phase orientation differences across platforms may affect image interpretation, especially when distinguishing paramagnetic from diamagnetic substances. While a vendor-specific stratification was beyond the scope of this study, it may be relevant for future multicenter or cross-platform analyses.

In times of increasingly high-resolution MRI examinations, there are also new possibilities for visualizing vascular structures that previously remained undetected. Our analysis specifically examined the “classical” DVA delineated by Lasjaunias and colleagues [16]. Ultra-high-field SWI at 7 Tesla MRI has unveiled additional atypical venous structures consistently associated with sporadic CCMs, and it is crucial to note that these structures should not be confused with the classical DVAs described earlier [6].

Our study meticulously adhered to rigorous methodological standards, including blinded assessments by experienced, board-certified neuroradiologists and comprehensive statistical analyses. Data were obtained from a sizable patient registry for CCMs, encompassing both treatment-naive sporadic and familial cases, thus reflecting the diversity within the underlying disease population. Nevertheless, certain limitations must be acknowledged. Firstly, the study’s retrospective nature and reliance on a single institutional database may introduce selection bias and limit generalizability. Additionally, while adequate for the study’s aims, the sample size may not fully capture the diversity of CCM and/or DVA cases, potentially influencing the observed diagnostic accuracy. Moreover, although we have defined CE-T1 sequences as the gold standard, this modality also carries the risk of false positive or negative findings. At this point, the case report by Chakrabarti and colleagues is worth mentioning, who point out that there are DVAs exclusively seen in hemosiderin-sensitive sequences but not in CE-T1 sequences [4]. This inherent problem might reduce the significance of our data. In light of this, the use of a multimodal imaging standard - combining CE-T1 with susceptibility-weighted sequences - may provide a more comprehensive basis for DVA detection. Such an approach could refine the estimation of sensitivity and specificity and better account for subtle or atypical DVA morphologies. While this was beyond the scope of our current study, it represents an important consideration for future investigations. Additionally, it’s important to acknowledge that the quality of MRI sequences has evolved significantly over the past two decades. As a result, older images may exhibit less optimal results compared to newer ones. Lastly, it is important to note that SWI and GRE-T2* sequences were obtained from different patient groups. As a result, any direct comparison of their diagnostic performance must be interpreted with caution. Differences in patient characteristics, pathology distribution, and imaging conditions may confound the observed results. While the findings provide valuable preliminary insights, further studies using both sequences in the same patient cohort are needed to enable a more robust and direct comparison.

The study’s findings have significant clinical implications. While CE-T1 imaging remains the gold standard for CCM and DVA detection, the reliance on gadolinium-based contrast agents poses potential risks, particularly in vulnerable patient populations [5]. Alternative imaging techniques such as SWI and GRE-T2* sequences offer non-invasive alternatives but should be interpreted cautiously, considering their inherent limitations. Clinicians must weigh the benefits of contrast-free imaging against the diagnostic accuracy required for surgical planning and patient management. Future research directions may include MRI studies on CCM to validate our findings across diverse patient populations and imaging platforms. In this context, a multicenter longitudinal study that directly compares CE and non-CE imaging approaches would be ideal to define optimal protocols for both lesion detection and follow-up. Additionally, advancements in imaging technology, such as machine learning algorithms for image analysis, may enhance the diagnostic capabilities of non-contrast MRI sequences [20, 21]. Besides exploring alternative imaging modalities, investigating combined approaches with SWI and GRE-T2* sequences may improve DVA detection accuracy, especially in challenging cases.

## Conclusions

In conclusion, while SWI and GRE-T2* sequences show promise as non-contrast alternatives for detecting CCM-associated DVAs, their diagnostic accuracy remains inferior to CE-T1 imaging. Clinicians should exercise caution when relying solely on these sequences for DVA detection, particularly in cases requiring surgical lesion removal. Further research and technological innovations are warranted to optimize non-contrast MRI techniques and improve diagnostic accuracy in CCM disease management.

## Electronic supplementary material

Below is the link to the electronic supplementary material.

Supplementary Material 1

## Abbreviations

CCM: Cerebral cavernous malformation
DVA: Developmental venous anomaly
GRE: Gradient echo imaging
SWI: Susceptibility weighted imaging
