# Supplementary material for: Detection of cerebral cavernous malformation associated developmental venous anomalies in gradient-echo and susceptibility-weighted magnetic resonance imaging: can we skip the contrast?
Source: Neuroradiology. 2025 Jun 13;67(8):2005–13. doi: 10.1007/s00234-025-03666-2 (PMC12494666; doi:10.1007/s00234-025-03666-2)
Supplement: Supplementary file 1 — Supplementary Material 1 [file 234_2025_3666_MOESM1_ESM.docx]

**Supplemental Fig 1 Detailed rating results of SWI and GRE-T2* sequences** For each sequence, the two groups [-DVA] (left) and [+DVA] (right) are shown. Each of the groups comprises 50 randomly selected cases. The first column shows the affiliation to the respective group, measured by the CE-T1 sequence. The second column shows the results according to Rater#1 and the third column shows the results according to Rater#2

|  | **+ DVA** | **- DVA** | **p** | **OR** | **95% CI** |
| --- | --- | --- | --- | --- | --- |
| No of patients (N, %) | 100 (100.0) | 100 (100.0) | N/A | N/A | N/A |
| Age (years, SD) | 43.7 (± 12.5) | 42.6 (± 16.9) | .58 ^A^ | N/A | N/A |
| Sex (N, %)  − Female − Male | 59 (59.0)  41 (41.0) | 49 (49.0)  51 (51.0) | .20 ^B^ | .67 | .38 – 1.17 |
| CCM multiplicity (N, %)  − 1 CCM  − ≥ 2 CCM | 72 (72.0)  28 (28.0) | 71 (71.0)  29 (29.0) | .99 ^B^ | .95 | .52 – 1.76 |
| CCM volume (cm^3^) | 4.60 (± 3.04) | 5.68 (± 5.59) | .09 ^A^ | N/A | N/A |
| CCM side ^C^ (N, %)  − Left  − Right | 50 (52.6)  45 (47.4) | 47 (49.5)  48 (50.5) | .77 ^B^ | 1.14 | .64 – 2.01 |
| CCM localization (N, %)  − Frontal lobe  − Temporal lobe  − Parietal lobe  − Occipital lobe  − Basal ganglia/thalamus  − Ventricular system  − Cerebellum  − Brainstem | 18 (18.0)  18 (18.0)  8 (8.0)  6 (6.0)  11 (11.0)  1 (1.0)  22 (22.0)  16 (16.0) | 24 (24.0)  19 (19.0)  8 (8.0)  7 (7.0)  6 (6.0)  1 (1.0)  8 (8.0)  27 (27.0) | .39 ^B^  .99 ^B^  .99 ^B^  .99 ^B^  .31 ^B^  .99 ^B^  **.01 ^B^**  .08 ^B^ | .70  .94  1.00  .85  1.94  1.00  3.24  .52 | .35 – 1.38  .46 – 1.91  .36 – 2.78  .28 – 2.62  .69 – 5.46  .06 – 16.21  1.37 – 7.69  .26 – 1.03 |
| CCM depth (N, %)  − Superficial  − Deep | 18  82 | 37  63 | **.00 ^B^** | 2.68 | 1.39 – 5.14 |
| **Supplemental Table 1 Characteristics of patients and cavernomas** Abbreviations: CCM, cerebral cavernous malformation; CI, confidence interval; DVA, developmental venous anomaly; N, number of patients; N/A, not applicable; No, Number; OR, odds ratio; SD, standard deviation. Annotations: A, t-test; B, chi-square test or Fisher test, where applicable; C, note that cavernomas of the midline are not considered here due to their rarity | | | | | |
